# Anticancer Activity of Enantiomeric Neplanocins A: Exploring the Role of Chirality in Tumor Suppression

**DOI:** 10.3390/ijms26031308

**Published:** 2025-02-04

**Authors:** Roza Pawlowska, Hubert Banaszkiewicz, Arkadiusz Chworos, Remigiusz Żurawiński

**Affiliations:** Centre of Molecular and Macromolecular Studies, Polish Academy of Sciences, Sienkiewicza 112, 90-363 Lodz, Poland; hubert.banaszkiewicz@cbmm.lodz.pl (H.B.); arkadiusz.chworos@cbmm.lodz.pl (A.C.)

**Keywords:** neplanocin A, adenosine analogue, chirality, adenosine kinase, adenosine deaminase, S-adenosylhomocysteine hydrolase, molecular docking

## Abstract

Neplanocin A (NPA) is a natural carbocyclic analogue of adenosine that was isolated from *Ampullariella regularis*, which is known for its antibacterial, antiviral, and anticancer activity. Although the activity of this compound has been demonstrated in many biological models, the mechanism of its anticancer activity is not fully understood. In the current work, we present the comparison of the biological activity of two enantiomers of neplanocin A in the series of cancerous and non-cancerous cell types. In all tested cell lines, the compound with natural stereochemistry, (-)-NPA, was found to be more cytotoxic than its synthetic (+)-NPA derivative; however, sensitivity to neplanocins A varied between cell types. To determine possible reasons for the observed differences in individual cancer cell types, the expression level and effects of individual genes of adenosine-interacting enzymes were analyzed. Bioinformatic analysis of the interaction between (-)-NPA and (+)-NPA with major adenosine-interacting enzymes, such as adenosine kinase (ADK), adenosine deaminases (ADA and ADA2), and S-adenosylhomocysteine hydrolase (SAHH, AHCY), was performed. The molecular docking results revealed differences in the binding energy of the individual enantiomers of neplanocin A with the targets, which sheds new light on the mechanism of action of these adenosine analogues.

## 1. Introduction

**Figure 1:**
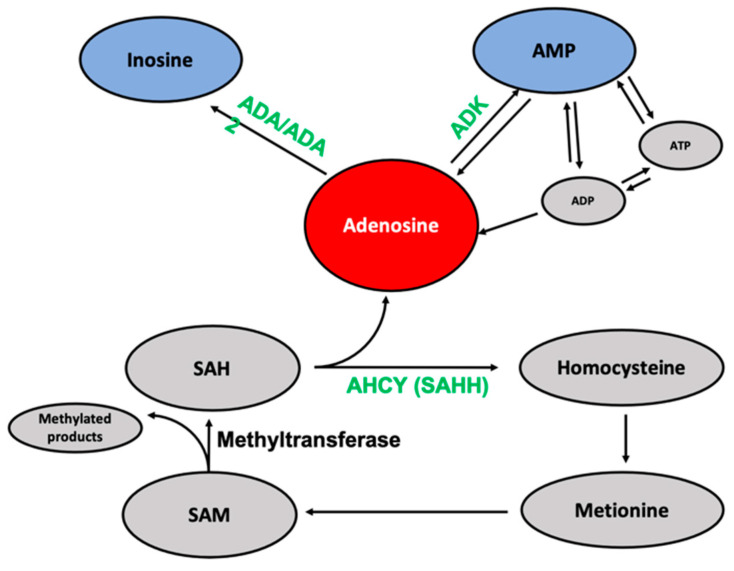
The most important cellular interactions of adenosine.

Adenosine is one of the basic nucleosides present both intra- and extracellularly, which exhibit an anticancer and antioxidant activity [1,2,3,4] and may act as a regulator of the immune system [5]. The activity of adenosine in cells is mainly related to its interaction with specific cellular targets such as adenosine kinase (ADK) and adenosine deaminases (ADA and ADA2), which convert it into AMP and inosine, respectively (Figure 1). As a precursor of ATP, adenosine plays a crucial role in ATP-dependent signaling pathways [6] and RNA synthesis [7].

**Figure 2:**
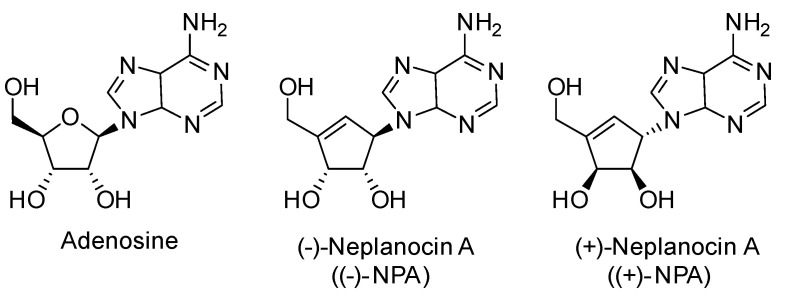
Structures of adenosine and enantiomeric neplanocins A.

Due to the wide range of adenosine activity, its derivatives have garnered significant interest as potential therapeutic agents. Adenosine analogues have been explored for a variety of biomedical applications [8,9], including antiproliferative [10] and antioxidant [2] treatments. This class of compounds is being tested as inhibitors of adenosine-binding enzymes [11], ligands for adenosine receptors [8,12,13], or precursors for modified ATP generation and further synthesis of therapeutic RNA [7]. Neplanocin A (9-[*trans*-2,*trans*-3-dihydroxy-4-(hydroxymethyl)cyclopent-4-enyl]adenine, NPA), a member of the carbocyclic nucleoside family, is a natural analogue of adenosine where the traditional sugar moiety is replaced by a carbocyclic unit (Figure 2).

The history of NPA dates back to 1981, when its levorotatory enantiomer ((-)-NPA) was first isolated from the culture filtrate of the soil-dwelling fungus *Ampullariella regularis* [14,15]. This discovery marked the beginning of extensive research into its biological properties and potential therapeutic applications of such an adenosine analogue. The modification of adenosine’s sugar framework resulted in the unique biological activity of natural (-)-NPA, including its broad antiviral [16,17,18] and anticancer properties [19,20,21,22,23]. These promising properties have driven significant synthetic efforts of many research groups, leading to the development of numerous means of the total synthesis of (-)-NPA [16,19,20,24,25,26,27,28,29,30,31,32,33,34,35,36,37,38,39,40,41,42,43,44,45]. In contrast, far fewer efforts have been directed toward synthesizing its enantiomeric analogue (+)-NPA, with only five total syntheses reported to date [16,19,46,47,48]. Although natural (-)-neplanocin A has been known for years, its mechanism of action has not been fully understood yet. Performed studies have revealed the inhibitory activity of (-)-NPA towards S-adenosylhomocysteine hydrolase (SAHH or AHCY), which catalyzes the hydrolysis of S-adenosylhomocysteine (SAH or AdoHcy) to adenosine and homocysteine, thereby demonstrating its participation in the regulation of AdoHcy levels inside the cells and cellular RNA methylation [49]. Inhibition of S-adenosylhomocysteine hydrolase results in AdoHcy accumulation [11] and disruption of methyl group donation to a variety of cellular targets [21,22]. Post-translational modifications such as histone methylation play a key role in gene regulation. Any disruption of this process may lead to pathological conditions and diseases such as cancer. Up-regulation of histone methylation is, for instance, observed in cancer cells. It was shown that inhibition of S-adenosylhomocysteine hydrolase by neplanocin A analogues may induce cytotoxic effects in cancer cells [21,22]. This effect was indicated to be related to the inhibition of the K79 lysine side chains methylation of histone H3 (H3K79) [21] or the H3K27 methyltransferase EZH2 [22]. However, numerous data show the possibility of the existence of SAHH-independent activity of neplanocin A and its analogues [11,50,51].

Interestingly, despite structural similarities, the activity of (-)-NPA and (+)-NPA differs. Differences were observed in both antiviral and anticancer studies. While (-)-NPA is effective against HIV-1 (EC₅₀ = 0.1 μM) and West Nile virus (EC₅₀ = 51 μM), (+)-NPA demonstrates significantly lower activity, being approximately 1000 times less active against HIV-1 and 4 times less active against the West Nile virus [16]. A similar trend is observed in cancer cells. The (-)-NPA is effective against MOLT-4 (IC₅₀ = 7 μM) and A431 (IC₅₀ = 10 μM) cancer cell lines, while (+)-NPA shows substantially reduced efficacy, with IC₅₀ values of 500 μM against MOLT-4 and 330 μM against A431, respectively [19]. These observations prompted us to conduct an in-depth analysis of the activity of individual NPA enantiomers in different cell types. To gain a clearer understanding of the factors contributing to the significant differences in activity between (-)-NPA and (+)-NPA, we selected cells with varying origins and expression profiles. For the assessment of the possible interaction with adenosine-binding enzymes, bioinformatic analysis was performed. The obtained data provided valuable knowledge for a more detailed investigation of the possible mechanism of activity of natural (-)-NPA.

## 2. Results

### 2.1. Cytotoxicity of (-)-Neplanocin A and (+)-Neplanocin A in Various Types of Cancer and Non-Cancerous Cells

**Figure 3:**
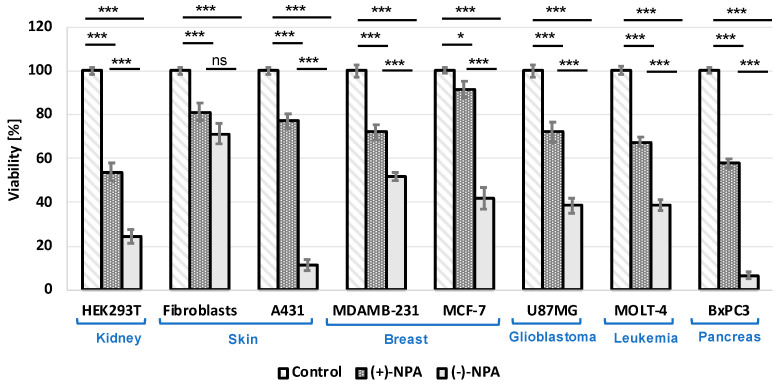
The viability of selected cell types after 48 h of incubation with 100 μM (+)-NPA and (-)-NPA. Viability values were calculated based on the MTT assay. Results for the cells treated with 100 μM (+)-NPA or (-)-NPA were calculated relative to the control samples (untreated cells) taken as 100%. All data are expressed as the mean of at least three independent experiments performed in triplicate ± SE. Statistical significance was determined using Student’s *t*-test; * *p* < 0.05, *** *p* < 0.0001, ns—not significant.

To compare the cytotoxicity of enantiomeric neplanocins A in human cells, a series of cancerous and non-cancerous cell lines were treated for 48 h with both enantiomers at a concentration of 100 µM. The differences in viability rate were observed for all types of tested cells, including both cancerous and non-cancerous cell types. In all cell types, the levorotatory enantiomer was more cytotoxic compared to its dextrorotatory counterpart (Figure 3); however, the differences varied depending on the cell type.

A large difference in survival rate after (+)-NPA and (-)-NPA treatment was observed for epidermoid carcinoma A431 cells, which was consistent with the previous report [19]. The viability rate of A431 cells treated for 48 h with 100 μM NPA-(-), it was 11%, while for cells treated with (+)-NPA, it reached 77%. Interestingly, for other non-cancerous cells—human fibroblasts—the viability of cells treated with both enantiomers, (-)-NPA and (+)-NPA, was very high at 71% and 81%, respectively. A difference in cytotoxicity between the individual enantiomers was also observed in the ER-positive MCF-7 breast cancer cells: only the (-)-NPA enantiomer showed cytotoxicity, resulting in 42% cell viability after 48 h of incubation, whereas the viability rate with (+)-NPA treatment was comparable to the control (91%). This is particularly interesting when compared to another breast cancer cell type—triple negative MDA-MB-231 cells—where the difference in cytotoxicity between (+)-NPA and (-)-NPA was less pronounced, with 72% and 52% cell viability, respectively, after 48 h of incubation.

### 2.2. Sensitivity of MDA-MB-231 Cells to Neplanocin A Under Different Conditions

It has been shown that the natural enantiomer of neplanocin A ((-)-NPA) can reduce the viability of triple-negative breast cancer (TNBC) cells [22,52] and affect their migration [52]. Therefore, it was plausible that (-)-NPA may also affect processes related to metastasis, such as epithelial-mesenchymal transition or anoikis resistance, in these highly metastatic cells. However, the influence of neplanocin A on the processes related to the aggressiveness of TNBC cells, such as resistance to anoikis, has not been demonstrated. Therefore, we decided to test the metastatic potential of this type of cancer cell in the presence of neplanocin A under different conditions.

Since (-)-NPA has been shown to induce apoptosis in cancer cells [19,52], it was reasonable to verify whether this analogue of adenosine may also sensitize cells to anoikis. The MDA-MB-231 was chosen as a model TNBC cell line. Anoikis, a particular kind of apoptotic cell death related to cell detachment from the extracellular matrix, was evaluated by measuring cell viability in ultra-low attachment plates. Such conditions, which prevent cells from attaching to the surface, allowed for the estimation of the anoikis resistance of the cells. Obtained data showed that neplanocin A did not affect the viability of MDA-MB-231 cells under these conditions and, therefore, did not reduce the anoikis resistance of this cell type. No reduction in anoikis resistance was observed following treatment with either enantiomer of neplanocin A under the tested conditions.

**Figure 4:**
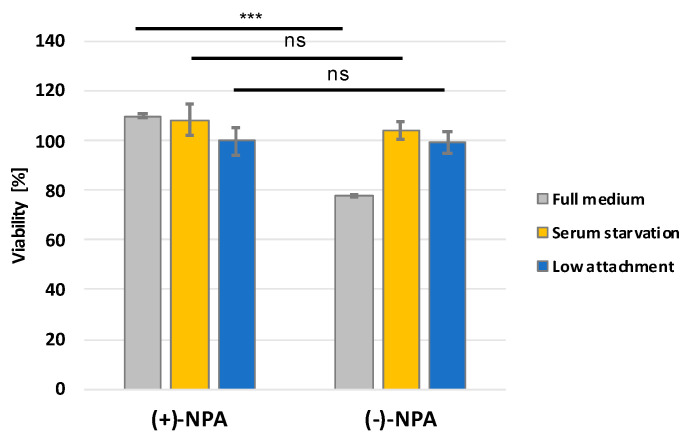
The influence of neplanocin A enantiomers on the viability of MDA-MB-231 cells under different cell culture conditions. MDA-MB-231 cells were incubated for 24 h with 10 μM (+)-NPA or (-)-NPA on ultra-low attachment (blue bar) or standard plates under serum-free (yellow bars) or full medium (grey bars) conditions. Each value represents the mean viability of cells treated with the respective NPA enantiomer ((+)-NPA or (-)-NPA) calculated relative to untreated cells cultured under the same conditions (taken as 100%). Statistical significance was determined using Student’s *t*-test; *** *p* < 0.0001, ns—not significant.

Interestingly, in a low-attachment environment, the cytotoxicity of (-)-NPA towards MDA-MB-231 cells was abolished overall (Figure 4), which could be related to the lower proliferation rate of the cells under such unfavorable conditions. A similar effect was noticed after serum starvation. Our results showed that whereas in the standard full medium, the 10 μM (-)-NPA treatment resulted in the decrease of viability of MDA-MB-231 cells by more than 20% after 24 h of incubation, serum starvation abolished the cytotoxicity of neplanocin A (Figure 4). Such an observation may be a result of the lower proliferation rate of cells under adverse conditions. Obtained results indicate that (-)-NPA acts mainly on highly proliferating cells, which is in accordance with previous observations. Under the same conditions, no cytotoxic effect was observed for (+)-NPA.

### 2.3. Analysis of Possible Interaction Partners for Neplanocin A in Cancer Cell Lines

The (-)-neplanocin A is a natural carbocyclic analogue of adenosine, and as such, it may be able to interact with adenosine-related enzymes (Figure 2) [53]. The main cellular targets for adenosine include adenosine kinases (ADK), adenosine deaminases (ADAs) [53], and S-adenosylhomocysteine hydrolase (AHCY, SAHH) [54] (Figure 1).

**Figure 5:**
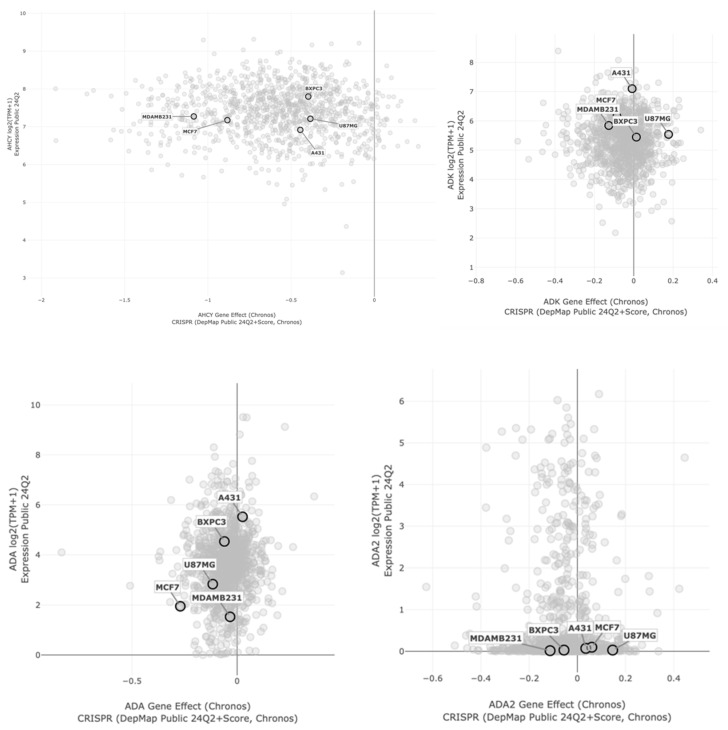
Analysis of cell-type-specific correlation between gene expression and effect of AHCY, ADK, ADA, and ADA2 in tested cancer cell lines.

To assess the potential of individual adenosine-interacting proteins in individual cell lines, we have analyzed the expression level and importance of its gene in cancer cell types used. Analysis was performed using the DepMap portal database (Figure 5).

The correlation between gene expression and its effect was plotted per tissue type. Based on the viability measurements, where A431 and BxPC3 cells were found to be the most sensitive to (-)-NPA, we particularly focused on the differences in these cell lines compared to others. Based on the literature data, the natural enantiomer of neplanocin A acts as an inhibitor of S-adenosylhomocysteine hydrolase (AHCY, SAHH); thus, it may be expected that its activity should be correlated with its expression level in particular cell lines. Although the expression level of AHCY did not correlate with the cytotoxicity results, the Chronos analysis divided tested cells into two groups, which partially correlated with the (-)-NPA cytotoxicity pattern (Figure 3).

Obtained data indicate that among adenosine-interacting enzymes, not only AHCY, but also adenosine kinase (ADK) and adenosine deaminase (ADA), deserve special attention as potential targets for neplanocin A. In both cases, the highest expression values were observed for (-)-NPA-sensitive A431 cells.

### 2.4. Bioinformatic Analysis of Neplanocin A Enantiomers with Adenosine-Related Cellular Targets

To estimate the possibility of interaction of neplanocin A enantiomers with adenosine-related targets, molecular docking analysis was performed. Obtained data indicate that both enantiomers of neplanocin A may interact with adenosine-dependent enzymes (Table 1).

**Figure 6:**
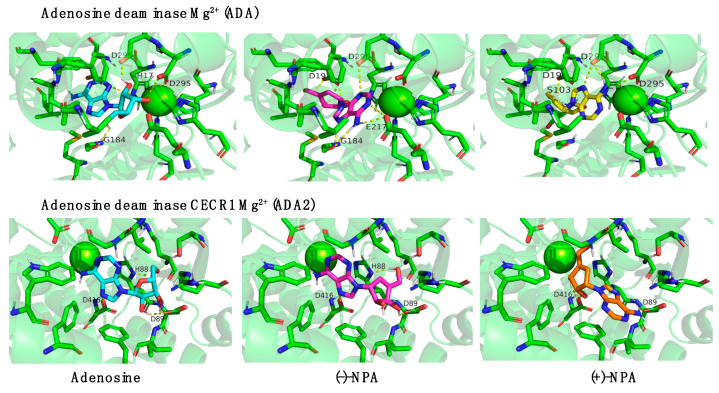
The molecular docking results of (-)-NPA and (+)-NPA interaction in the active center of adenosine deaminases (ADA and ADA2) using Mg^2+^ ions. The numbers of amino acids were assigned with the FASTA sequence of protein taken from the UniProt database as a base template.

Since it was demonstrated that (-)-NPA is an inhibitor of S-adenosylhomocysteine hydrolase, we have evaluated the possible interaction of the dextrorotatory enantiomer of neplanocin A. The binding energy of (+)-NPA to AHCY (−12.22 ± 0.00) occurred to be similar to the values observed for (-)-NPA (−12.29 ± 0.03) and adenosine (−12.18 ± 0.03), which was unexpected, since the (-)-NPA enantiomer was selected as an active inhibitor. This result indicates that the (+)-NPA can form equally strong complexes with the enzyme as (-)-NPA. Thus, it can be assumed that there may also exist AHCY-independent factors that contribute to the differences in the biological activity of the neplanocin A enantiomers. Due to their structural similarity, the most likely interaction partners for these types of compounds seem to be adenosine-binding enzymes. Based on the fact that the natural cellular target proteins for interaction with adenosine are adenosine kinase (ADK) and adenosine deaminases (ADA and ADA2) (Figure S1) further bioinformatic analysis was focused on these enzymes. Molecular docking of adenosine and both enantiomers of NPA was performed with ADA and ADA2 in several configurations: with Zn^2+^ (Figure S2) as it is a natural cofactor, based on the original crystal structure and with Mg^2+^ (Figure 6). Complex structures between Ade and both NPAs with selected enzymes using Mg^2+^ ions had slightly stronger binding energies. Among these ligands, higher binding energies were observed for neplanocin A enantiomers compared to the natural ligand, adenosine. These results were obtained for both adenosine deaminases, which suggests that (-)-NPA and (+)-NPA may interact with this class of enzymes.

**Figure 7:**
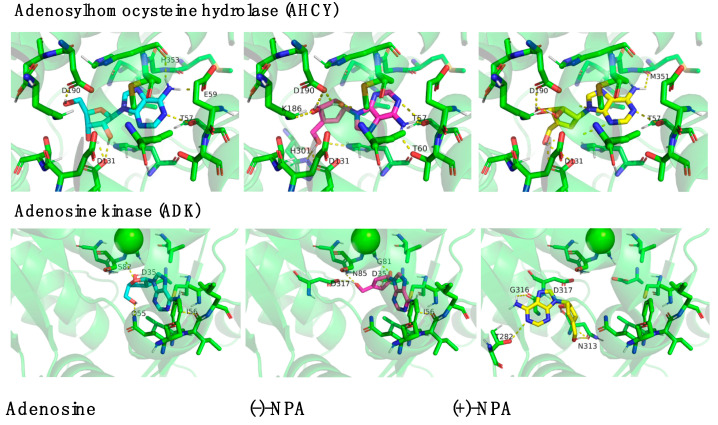
The molecular docking results of (-)-NPA and (+)-NPA interaction in the active center of S-adenosylhomocysteine hydrolase (AHCY) and adenosine kinase (ADK) compared to the adenosine. The numbers of amino acids were assigned with the FASTA sequence of the protein from the UniProt database.

The biggest difference between (-)-NPA and (+)-NPA in binding with the tested protein was obtained for adenosine kinase (Table 1, Figure 7 ADK). While the position of the (-)-NPA enantiomer in the active center of the enzyme was similar to that of its natural ligand, adenosine, maintaining interaction with Asp35 and Ile56, the location of (+)-NPA was somewhat different. It extended beyond the standard binding site, with only Asp317 interacting like (-)-NPA. Considering the differences in the localization of this derivative in the enzyme’s active site and its lower binding energy compared to the natural substrate, it may be assumed that this enantiomer may not be processed by the enzyme in the same way as native adenosine.

In the case of (-)-NPA, the binding energy with the adenosine kinase was over 1 kcal/mol higher (−10.75 kcal/mol) than for (+)-NPA (−9.71 kcal/mol) and the natural ligand (−9.83 kcal/mol) (Table 1). The differences in the ligand binding observed for individual enantiomers of neplanocin A suggest that ADK may play an important role in the biological activity of these carbocyclic analogues of adenosine; however, further research is needed for an in-depth analysis of the potential role of these interactions. This is especially important considering that adenosine, as a natural ligand, is processed by hydrolyzes (ADAs) and phosphorylated in the case of ADK. Neplanocin A enantiomers, although they have similar or higher binding energies, may not be processed the same way as a natural analogue, and thus (-)-NPA was selected as an inhibitor. In this case, the (+)-NPA enantiomer may appear as an alternative or even better inhibitor. What is particularly interesting, though not entirely surprising, is that S-adenosylhomocysteine hydrolase (AHCY), an enzyme that catalyzes the hydrolysis of S-adenosylhomocysteine (SAH), binds all three ligands with the highest binding energy (over −12 kcal/mol) (Table 1), even in the absence of any metal ions. Each ligand interacts with three amino acids—Asp190, Asp131, and Thr57—showing similar conformation. This enzyme does not require cofactors; however, in theoretical protein predictions, divalent ions often form favorable salt bridges in the structures. Here also both NPAs are somewhat better than natural adenosine. Also, the structure of the ligand in the active center is similar among all three analogues, with the (+)-NPA base almost identical to the one of adenosine (Figure 7 AHCY); ligand to ligand RMSD = 5.642.

## 3. Discussion

The mechanism of anticancer action of neplanocin A has not been fully elucidated yet; hence, it is reasonable to analyze the relationship between the structure of the molecule and its biological activity. In the current research, we present data demonstrating how the stereochemistry of neplanocin A may affect its binding to the biological targets and anticancer potential. Literature data shows that the stereochemistry of substrates may be crucial for their recognition by enzymes [9,55,56] and surface receptors [57]. It was shown that conformational changes in both parts of the nucleoside—in sugar (north or south) and nucleobase (*syn* or *anti*)—may contribute to the differences in their biological activity [51]. Certain carbocyclic nucleosides with D-like configuration, which is similar to naturally occurring nucleosides, such as abacavir or entecavir, might be able to interact with target enzymes [17], whereas the activity of their derivatives with L-configuration varies depending on the compound [51]. These examples underscore the critical role that chirality plays in biological activity, as often only one enantiomer achieves the desired therapeutic effects. This difference in activity largely arises from the specific interactions of each enantiomeric form with chiral biological targets, such as enzymes, transporters, and receptors.

Here we present data demonstrating differences in the cytotoxic activity of neplanocin A enantiomers: (-)-NPA and (+)-NPA for a series of cancerous (MDA-MB-231, MCF-7, A431, U87-MG, Bx-PC3, and MOLT-4) and non-cancerous (human fibroblasts and HEK293T) cells. In all cell types, the cytotoxicity of (-)-NPA was higher than for (+)-NPA, which is in accordance with the previous observations [19]. It was shown that (-)-NPA selectively affects A431 cells, which results in the induction of apoptosis [19]. Programmed cell death initiation with analogues of neplanocin A and 3-deazaneplanocin A was also demonstrated in colon cancer HCT116 [58] and in triple-negative breast cancer cells MDA-MB-231 [22]. Additionally, fluoro-neplanocin A derivatives were shown to decrease the migration rate of these breast cancer cells [52]. Based on these results, we have hypothesized that neplanocin A treatment might affect the anoikis resistance of these highly metastatic cancer cells, but the studies performed did not reveal statistically significant differences in the anoikis resistance of cells treated with 10 μM neplanocin A enantiomers. Furthermore, serum starvation conditions abolished the cytotoxic effect of (-)-NPA, supporting the hypothesis that the neplanocin A activity is mainly related to highly proliferating cells.

It was also shown that natural (-)-NPA suppressed RNA synthesis [19,23] in certain cancer cells; however, the exact mechanism underlying its biological activity remains unresolved. Numerous data showed the S-adenosylhomocysteine hydrolase (AHCY, SAHH), which catalyzes the interconversion of S-adenosylhomocysteine (SAH) into adenosine and L-homocysteine, is a target for (-)-NPA [20,49]. It was demonstrated that (-)-NPA inhibits SAHH and therefore is involved in the regulation of the level of cellular homocysteine and methylation status of SAHH-dependent targets [21,49]. For example, the anti-proliferating effect of (-)-neplanocin on the MDA-MB-231 cells was shown to be connected with the inhibition of histone H3K79 methylation [21].

However, it was also shown that the antibacterial, antiviral, and anticancer activity of neplanocin A may also be independent of the inhibition of SAHH [11,50,51]. Thus, it may be assumed that the existence of an alternative mechanism of neplanocin A activity is possible. Studies using various neplanocin A analogues shed some light on possible interactions and signaling pathways in which neplanocin A may be involved.

Some data indicate that a key point in neplanocin A activity may be its phosphorylation by adenosine kinase (ADK) [23]; however, one of the L-like derivatives of neplanocin—DHCDA(9-(*trans*-2′,*trans*-3′-dihydroxycyclopent-4′-enyl)-3-deazaadenine), lacking the 5′-hydroxyl group necessary for the 5′-phosphorylation reaction and simultaneously inactive for AdoHcy hydrolase—occurred to exhibit antiviral activity for a broad spectrum of viruses [51]. It was indicated that neplanocin A may act as a competitive inhibitor for adenosine kinase, but it should be noted that adenosine was not able to reverse the growth-inhibitory action of (-)-NPA [23].

Analysis of the expression level of genes coding adenosine-interacting proteins and their effect on the cancer cells, conducted using the DepMap portal database, indicated that other than the AHCY-dependent mechanism of action of neplanocin A, it is possible. To verify whether the 5′-hydroxy group in (-)-NPA and (+)-NPA can be phosphorylated in a similar manner as in adenosine, the molecular docking with adenosine kinase was performed. Obtained results revealed potential selectivity of this enzyme for individual enantiomers of neplanocin A, which correlates with cytotoxicity measurements and gene expression and effect analysis. The high expression and cellular effect in the (-)-NPA-sensitive A431 cells, compared to the highest binding energy, make adenosine kinase (ADK) a potent and important target for (-)-NPA; however, further research is needed to prove it. Numerous data indicated the involvement of adenosine kinase in carcinogenesis and epigenetic regulation of pro-angiogenic factors. ADK downregulation was shown to suppress proliferation, viability, migration, and invasion of cancer cells [53].

Furthermore, interesting data were obtained for the unnatural enantiomer of neplanocin A. Literature data showed that the activity of S-adenosylhomocysteine hydrolase is comparable in HEK-293 and MCF-7 cells [59], thus it may be expected that treatment with AHCY-dependent compounds should lead to similar effects, whereas the sensitivity of these two types of cells for (+)-NPA varied. After 48 h of treatment with this enantiomer at a concentration of 100 µM, the viability of HEK-293 cells decreased to 54%, while the viability of MCF-7 cells remained similar to the control, at 91%. Such a large difference in cell viability with similar activity of the enzyme, compared with the bioinformatic analysis, indicated that (+)-NPA may interact with targets other than AHCY. Moreover, this cellular interaction seemed to be different than for (-)-NPA. Molecular docking results indicated adenosine deaminase as a possible target for neplanocin A. The binding energy for (+)-NPA with ADA and ADA2 was higher in both variants of the calculation compared to (-)-NPA and the natural ligand (adenosine), which supports this hypothesis. The changes in adenosine deaminase (ADA1 and ADA2) activity have been detected in various types of cancer cells, including breast, gastric, bladder, colon, and kidney cancer cells. Although the exact role of adenosine deaminases in cancer progression remains unclear, evidence suggests that these enzymes may regulate adenosine and inosine levels in cancer cells and the tumor microenvironment. Increased expression of adenosine deaminases can lead not only to a reduction in adenosine levels but also to the accumulation of inosine. Therefore, inhibitors of these enzymes are being tested as potential therapeutics for various diseases, including cancers [53].

In summary, the obtained data suggest that while both enantiomers of neplanocin A may interact with the cellular targets for adenosine—such as adenosine kinase (ADK), adenosine deaminases (ADA and ADA2), and S-adenosylhomocysteine hydrolase (AHCY)—the specificity of these interactions may differ between the enantiomers. Furthermore, our results indicate that the anticancer mechanism of action of natural neplanocin A ((-)-NPA) seemed to be connected with adenosine kinase activity, while the activity of its opposite enantiomer may be related to the interaction with adenosine deaminase; however, further investigation is needed to identify details of this dependence and to understand the exact signaling pathways leading to the observed effects.

## 4. Materials and Methods

### 4.1. Chemicals

Both enantiomers of Neplanocin A: (-)-NPA and (+)-NPA were synthesized according to the previously described procedure [19]. NMR spectra of enantiomeric neplanocins A were recorded on Bruker DRX 500 or Bruker AV Neo 400 spectrometers (Bruker, Billerica, MA, USA) and are included in the Supplementary Materials (Figures S3 and S4). Optical rotations were measured using a Perkin-Elmer MC 241 photopolarimeter. (+)-NPA: Mp 212–215 °C. [α]_D_^25^ = +153.0 (c 0.5 in H_2_O). ^1^H NMR (400 MHz, DMSO-*d*_6_): δ 8.12 (s, 1 H), 8.06 (s, 1 H), 7.20 (s, 2 H), 5.70 (s, 1 H), 5.34 (s, 1 H), 5.14 (d, *J* = 6.8, 1 H), 4.97 (d, *J* = 6.1, 1 H), 4.91 (t, *J* = 5.5, 1 H), 4.42 (d, *J* = 5.9, 1 H), 4.31 (q, *J* = 6.1), 4.11 (s, 2 H); ^13^C NMR (126 MHz, DMSO-*d*_6_) δ 156.03, 152.34, 150.08, 149.68, 139.59, 123.48, 119.22, 76.61, 72.25, 64.26, 58.59. (-)-NPA: Mp 214–217 °C. [α]_D_^25^ = −154.0 (c 0.4 in H_2_O).

### 4.2. Cell Culture

The research was performed using non-cancerous cells, including HEK293T and human fibroblasts, as well as cancer cell lines: A431 (squamous cell carcinoma), MDA-MB-231 (human breast adenocarcinoma), BxPC-3 (pancreatic adenocarcinoma), MCF-7 (breast cancer), U87-MG (human glioblastoma), and MOLT-4 (T-cell acute lymphoblastic leukemia).

The MDA-MB-231 (human breast adenocarcinoma) was purchased from Cell Biolabs (San Diego, CA, USA); MOLT-4 and U87-MG cells were purchased from the European Collection of Authenticated Cell Cultures (ECACC, Salisbury, UK); A431 was purchased from the American Type Culture Collection (ATCC, Manassas, VA, USA).

The fibroblasts, A431, MCF-7, and HEK293T cells were cultured in Dulbecco’s modified Eagle’s medium (DMEM) (Biological Industries) with 10% fetal bovine serum (FBS) (Sigma-Aldrich, St. Louis, MO, USA) and antibiotics (100 U/mL penicillin and 100 μg/mL streptomycin (BioWest, Lakewood Ranch, FL, USA)). The U87-MG cells were cultured in MEM medium (Sigma-Aldrich, St. Louis, MO, USA) containing 10% FBS (Sigma-Aldrich, St. Louis, MO, USA) and antibiotics (100 U/mL penicillin and 100 μg/mL streptomycin (BioWest)).

The MDA-MB-231 cells were cultured in DMEM medium (Biological Industries) with 10% FBS (Sigma-Aldrich, St. Louis, MO, USA), antibiotics (100 U/mL penicillin and 100 μg/mL streptomycin (BioWest)) supplemented with L-glutamine (Biological Industries) and non-essential amino acids (BioWest). BxPC-3 and MOLT-4 cells were cultured in RPMI medium (Biological Industries) with 10% fetal bovine serum (FBS) (Sigma-Aldrich, St. Louis, MO, USA) and antibiotics (100 U/mL penicillin and 100 μg/mL streptomycin (BioWest)).

Cells were passaged twice a week. Cultures were performed under standard conditions (37 °C, 5% CO_2_). Prior to the usage, cultures were tested for the presence of mycoplasma infection to ensure mycoplasma-free conditions. Tests were performed using the EZ-PCR Mycoplasma Test Kit with an internal control (Biological Industries, Izrael). For each experiment, cells were counted using the Scepter^TM^ 2.0 Cell Counter (Merck, Darmstadt, Germany).

### 4.3. Cytotoxicity Test

Cytotoxicity of compounds was assessed using the MTT test. For this purpose, cells were seeded into 96-well plates at 10,000 cells per well. After 24 h, the culture medium was removed, and a fresh medium containing tested compounds was added to the wells. Cells were cultured in the presence of tested compounds for 48 h. Culture was performed under standard conditions (37 °C, 5% CO_2_). After 48 h of incubation, the MTT reagent ((3-(4,5-dimethyl-2-thiazolyl)-2,5-diphenyltetrazolium bromide) was added to the wells. Cells were then incubated for 2 h at 37 °C. After this time, the culture medium was removed, and 100 µL of isopropanol was added to each well. Plates were shaken for 1 h at room temperature, and the absorbance was measured at 570 nm with 630 nm as a reference, using a Synergy HT plate reader (Bio-Tek, Winooski, Vermont, USA) and KC4 3.2 Rev 2 software (Bio-Tek, Winooski, Vermont, USA). The cytotoxicity level of each compound was calculated relative to the negative control (H_2_O or DMSO-treated cells for adenosine or enantiomers of neplanocin A, respectively). The viability of control cells was taken as 100%. Each viability point represents the mean value from at least three independent experiments performed in triplicate.

### 4.4. Anoikis Resistance Assay

For the anoikis resistance calculation, MDA-MB-231 cells were seeded into an Ultra Low Attachment 96-well plate (Corning, NY, USA) at a concentration of 10,000 cells per well in DMEM medium (Biological Industries) supplemented with L-glutamine (Biological Industries), non-essential amino acids (BioWest), and antibiotics (100 U/mL penicillin and 100 μg/mL streptomycin (BioWest)). Cells were incubated for 24 h in the presence or absence of tested neplanocin A enantiomers. As a control, identical experiments were carried out on standard Nunclon Delta Surface 96 well plates (Thermo Scientific, Denmark) in serum-free conditions and with a 10% addition of FBS (Sigma-Aldrich, St. Louis, MO). After 24 h of incubation, the viability test was performed on all plates. The viability was measured according to the procedure described in paragraph 4.3. Cells treated only with DMSO (without neplanocin A enantiomers) were used as a control.

### 4.5. Computational Analysis of Gene Expression and Its Effects on Cancer Cell Lines

The correlation between gene expression and gene effect in the selected cell lines was performed based on the DepMap portal (http://depmap.org/portal) [60] using data version Expression Public 24Q2 and CRISPR (DepMap Public 24Q2+Score, Chronos). Chronos was used as an algorithm for inferring gene fitness effects from CRISPR knockout experiments [61]. The following genes were analyzed: *AHCY*, *ADK*, *ADA,* and *ADA2*.

### 4.6. Statistical Analysis of Experimental Studies

All experimental data are presented as mean values of at least three independent experiments ± standard error. Individual variants were repeated at least three times in each experiment. The results for the tested compounds were calculated relative to the control samples (untreated cells). Statistical significance was analyzed using Student’s *t*-test. The analysis was performed using GraphPad Prism (San Diego, CA, U.S.A.). Differences were considered statistically significant at * *p* < 0.05, ** *p* < 0.01, and *** *p* < 0.0001.

### 4.7. Protein Structures Preparation for Docking Analysis

The FASTA sequences of ADA, ADA2, ADK, and AHCY native proteins were downloaded from the uniport server (ADA—[UniProt—P00813], ADA2—[UniProt—Q9NZK5], AD—[UniProt—P55263], AHCY—[UniProt—P23526]. The 3D structures of protein fragments along with the FASTA sequence of crystallized enzymes for those fragments were obtained from the Protein Data Bank (ADA PDB ID: 7RTG, ADA2 PDB ID: 3LGD, ADK PDB ID: 1BX4, AHCY PDB ID: 1LI4). The water molecules and ligands were removed from the structures. The number of chains in the structures varied depending on the protein: ADA and ADA2 had 2 chains, ADK had 1 chain, and AHCY had 4 chains. In the active centers of the proteins, different ions were present: Zn^2+^ for ADA and ADA2, Mg^2+^ for ADK, and no ions for AHCY. Structural alignment of chains existing in respected structures shows the negligible difference in the active center of the respected protein; based on that, in all of the proteins, chain A was chosen for further analysis. Next, the sequence alignment of the whole protein sequence (Uniport) with the fragments (Protein Data Bank). Then the structure was completed using Swiss-model (Figure S2).

### 4.8. Molecular Docking with AutoDock4

The adenosine, (-)-NPA, and (+)-NPA were selected as ligands for ADA, ADA2, ADK, and AHCY proteins. The adenosine structure was downloaded as a natural cofactor from PubChem (60961); (-)-NPA was also acquired from PubChem (137349804), and (+)-NPA was built based on the other enantiomer, followed by energy minimization and reconstruction of missing atoms with MGL Tools. Molecular docking with ADA and ADA2 was performed in 2 variants: (1) with Zn^2+^ ions as in the original crystal structure; (2) with Zn^2+^ substituted for Mg^2+^. Magnesium ions were selected as a substitute because native zinc ions are not well-parametrized for molecular docking. The Mg^2+^ ions, yet slightly smaller than Zn^2+^, are much better set for structural analysis, including molecular docking and molecular dynamics. For ADK, molecular docking was performed with Mg^2+^ as in the original crystal structure. For AHCY, molecular docking was performed without metal ions because none are present as natural cofactors of the protein. The model preparation was completed with MGL. Tools and molecular docking using Autodock 4.2.6. The set of parameters is as follows:

ADA: Grid center: x, y, z = 6.705, 10.745, 16.930: Grid Size x, y, z = 60: spacing 0.375

ADA2: Grid center: x, y, z = 9.740, 8.640, 18.265: Grid Size x, y, z = 60: spacing 0.375

ADK: Grid center: x, y, z = 47.347, 16.200, 38.408: Grid Size x, y, z = 60: spacing 0.375

AHCY: Grid center: x, y, z = 47.597, 18.698, 104.172: Grid Size x, y, z = 60: spacing 0.375

The 3D models of docked ligands and proteins were built using PyMol.

### 4.9. Statistical Analysis of Binding Energy Calculation

The average binding energy for adenosine and NPA isomers was calculated based on the 10 best structures selected from a series of two calculations, each generating 100 complexes. Each value represents a mean (μ) of the binding energy values calculated using the standard formula for the individual binding energy values. For instance, in the given dataset, the binding energy values were summarized and divided by the number of structures used for calculation. The standard error was then computed by the standard deviation, obtained by taking the square root of the mean of the squared differences. This method ensures that the standard deviation accurately reflects the dispersion of binding energy values around the mean. The computed standard deviation provides insight into the variability and consistency of the binding energy measurements within the dataset.

## Figures and Tables

**Table 1:** The average energy binding of (-)-NPA and (+)-NPA and adenosine with AHCY, ADA, ADA2, and ADK ± standard deviation.

Av, ΔΔG [kcal/mol]	Adenosine	(-)-NPA	(+)-NPA
AHCY (none)	−12.18 ± 0.03	−12.29 ± 0.03	−12.22 ± 0.00
ADA (Zn^2+^)	−10.45 ± 0.19	−10.09 ± 0.02	−11.09 ± 0.03
ADA (Mg^2+^)	−11.01 ± 0.03	−11.29 ±0.01	−11.41 ± 0.01
ADA2 (Zn^2+^)	−9.61 ± 0.04	−9.88 ± 0.27	−10.44 ± 0.08
ADA2 (Mg^2+^)	−9.65 ± 0.12	−10.02 ± 0.22	−10.84 ± 0.07
ADK (Mg^2+^)	−9.83 ± 0.10	−10.75 ± 0.10	−9.71 ± 0.05

## Data Availability

The data presented in this study are available in Supplementary Materials.

## Supplementary Materials

The following supporting information can be downloaded at: https://www.mdpi.com/article/10.3390/ijms26031308/s1.
