# Supplementary material for: Anticancer Activity of Enantiomeric Neplanocins A: Exploring the Role of Chirality in Tumor Suppression
Source: Int J Mol Sci. 2025 Feb 4;26(3):1308. doi: 10.3390/ijms26031308 (PMC11818473; doi:10.3390/ijms26031308)
Supplement: Supplementary file 1 [file ijms-26-01308-s001.zip › ijms-3341076-supplementary.pdf]

Supplementary Materials

Anticancer Activity of Enantiomeric Neplanocins A: Exploring the Role of Chirality in Tumor Suppression

Roza Pawlowska [1,*], Hubert Banaszkiewicz [1], Arkadiusz Chworos [1] and Remigiusz Żurawiński [1,*]

[1] Centre of Molecular and Macromolecular Studies, Polish Academy of Sciences, Sienkiewicza 112, 90-363 Lodz, Poland; roza.pawlowska@cbmm.lodz.pl (R.P.); hubert.banaszkiewicz@cbmm.lodz.pl (H.B.); arkadiusz.chworos@cbmm.lodz.pl (A.C.); remigiusz.zurawinski@cbmm.lodz.pl (R.Z.)

* Correspondence: roza.pawlowska@cbmm.lodz.pl (R.P.); remigiusz.zurawinski@cbmm.lodz.pl (R.Z.)

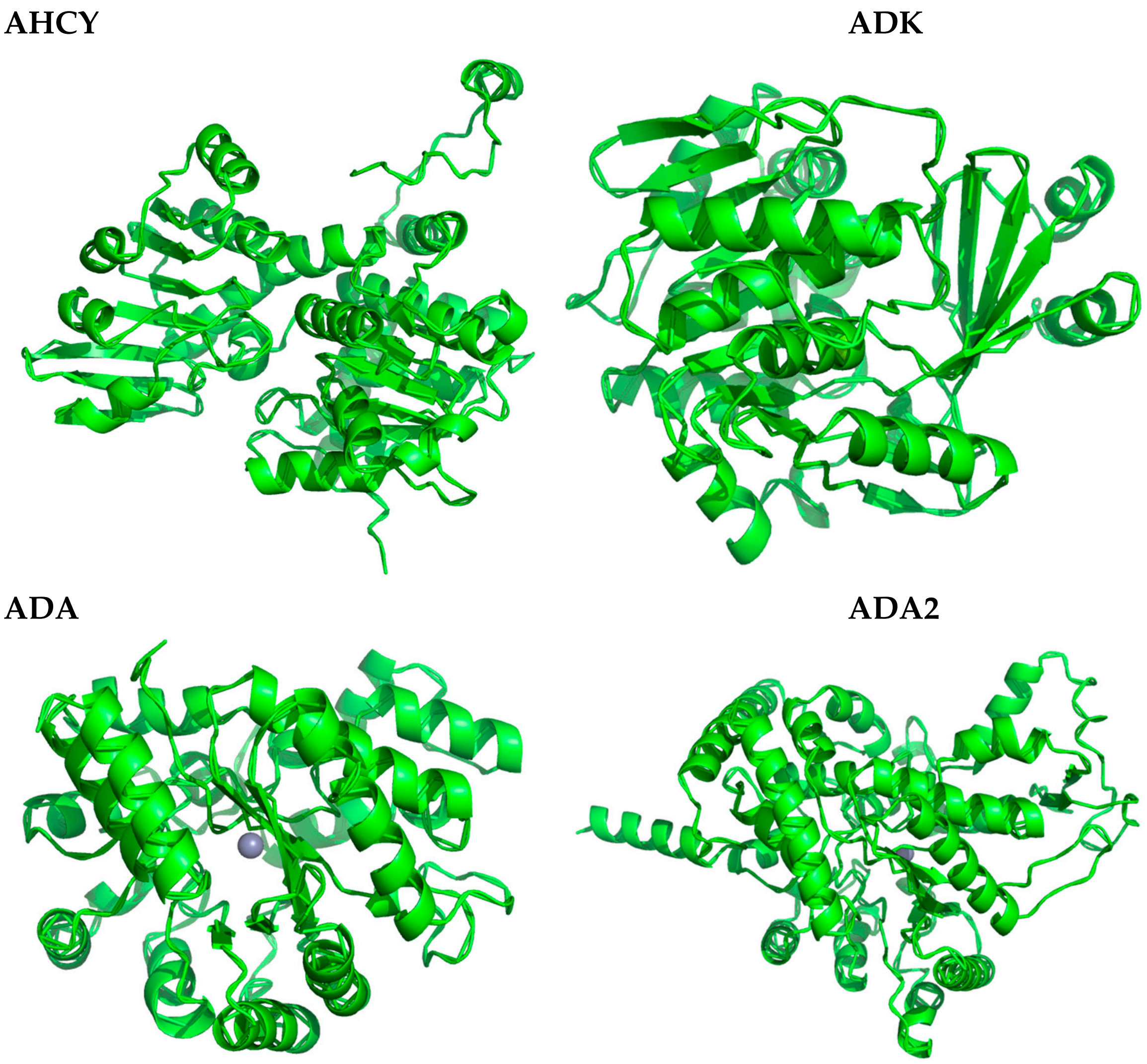

Figure S1. The protein structures used for molecular docking analysis.

Adenosine deaminase Zn^{2+} (ADA)

Adenosine deaminase CECR1 Zn^{2+} (ADA2)

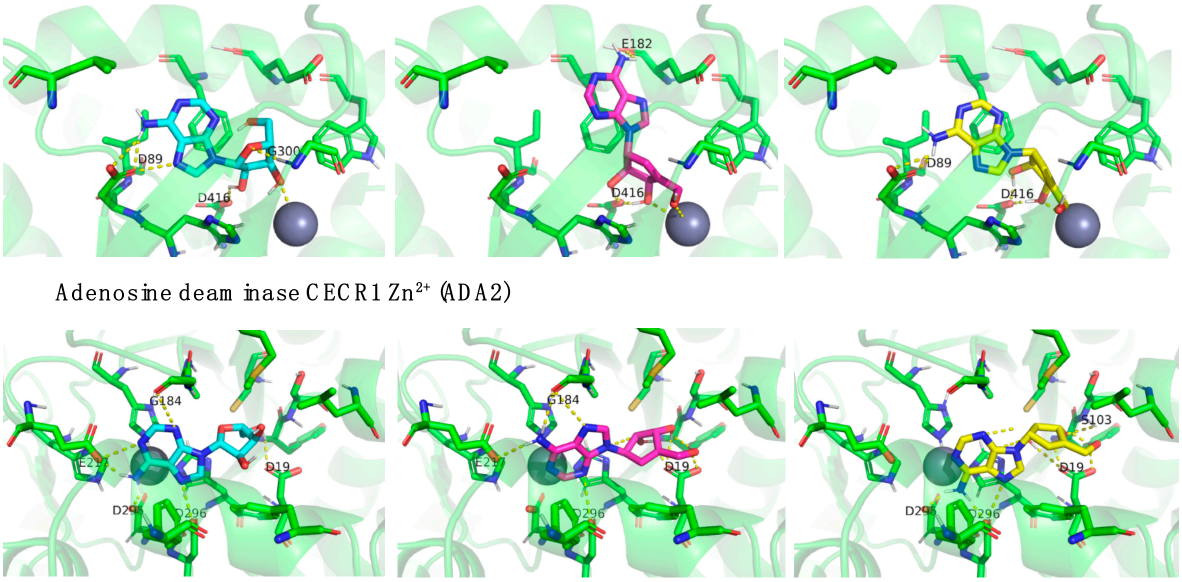

Adenosine (-)-NPA (+)-NPA

Figure S2. The molecular docking results of (-)-NPA and (+)-NPA interaction in the active center of adenosine deaminases (ADA and ADA2) using Zn^{2+} ions. The numbers of amino acids were assigned with FASTA sequence of protein taken from UniProt data base as base template.

NMR spectra of (-)-NPA and (+)-NPA

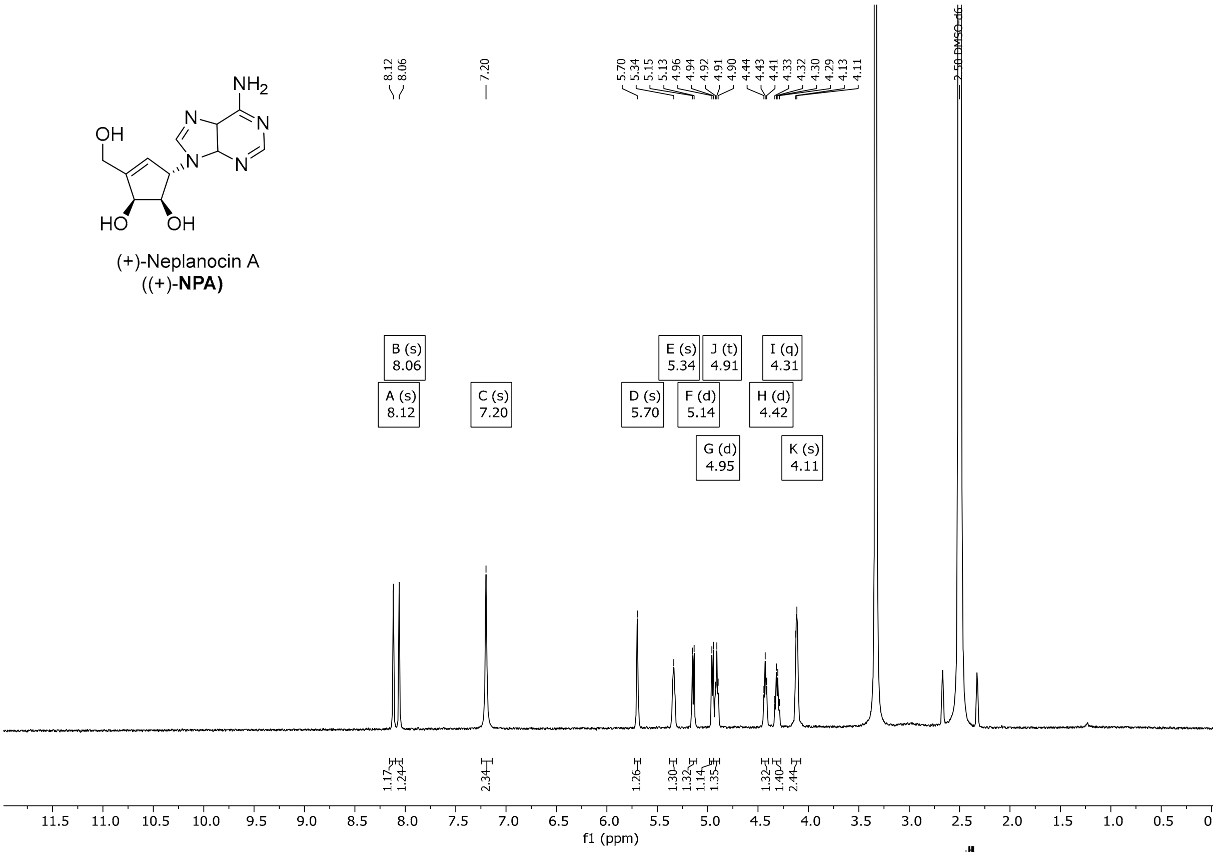

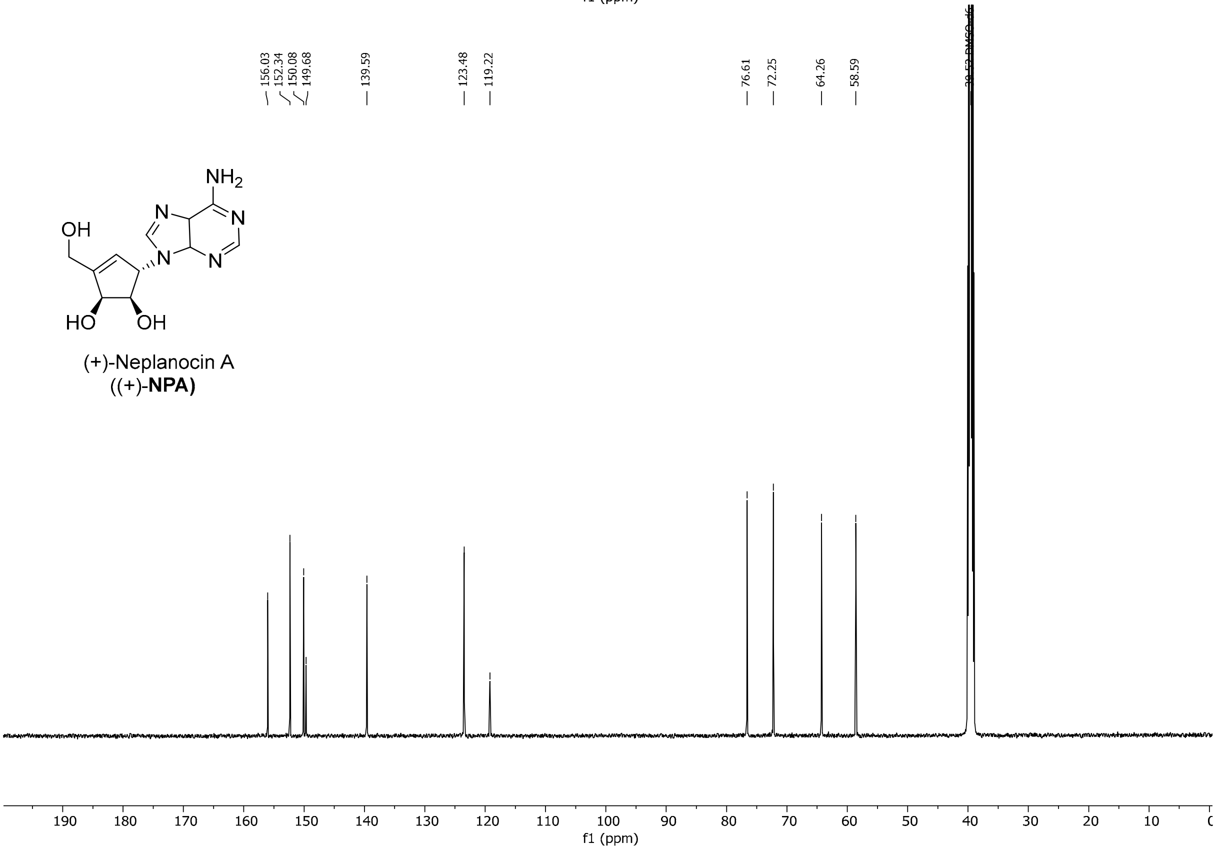

Figure S3. NMR spectra of (+)-NPA.

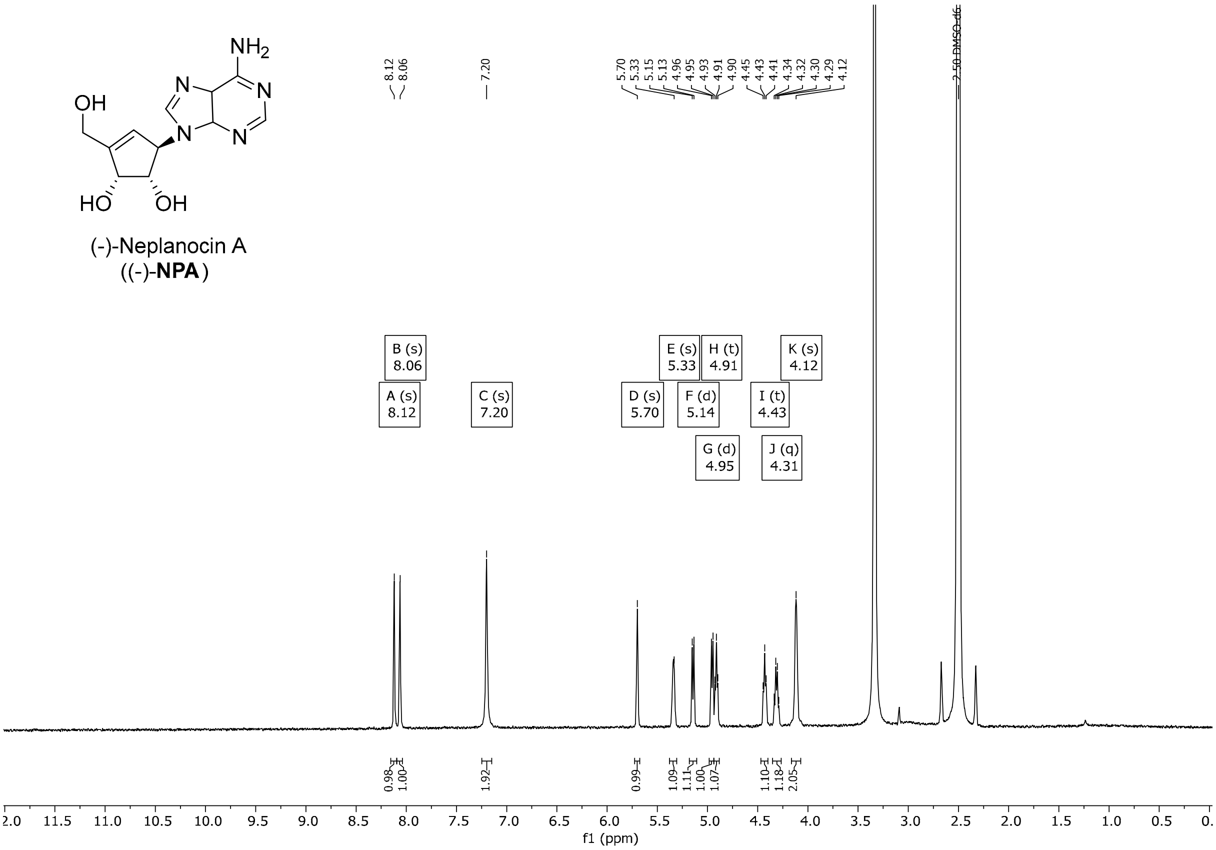

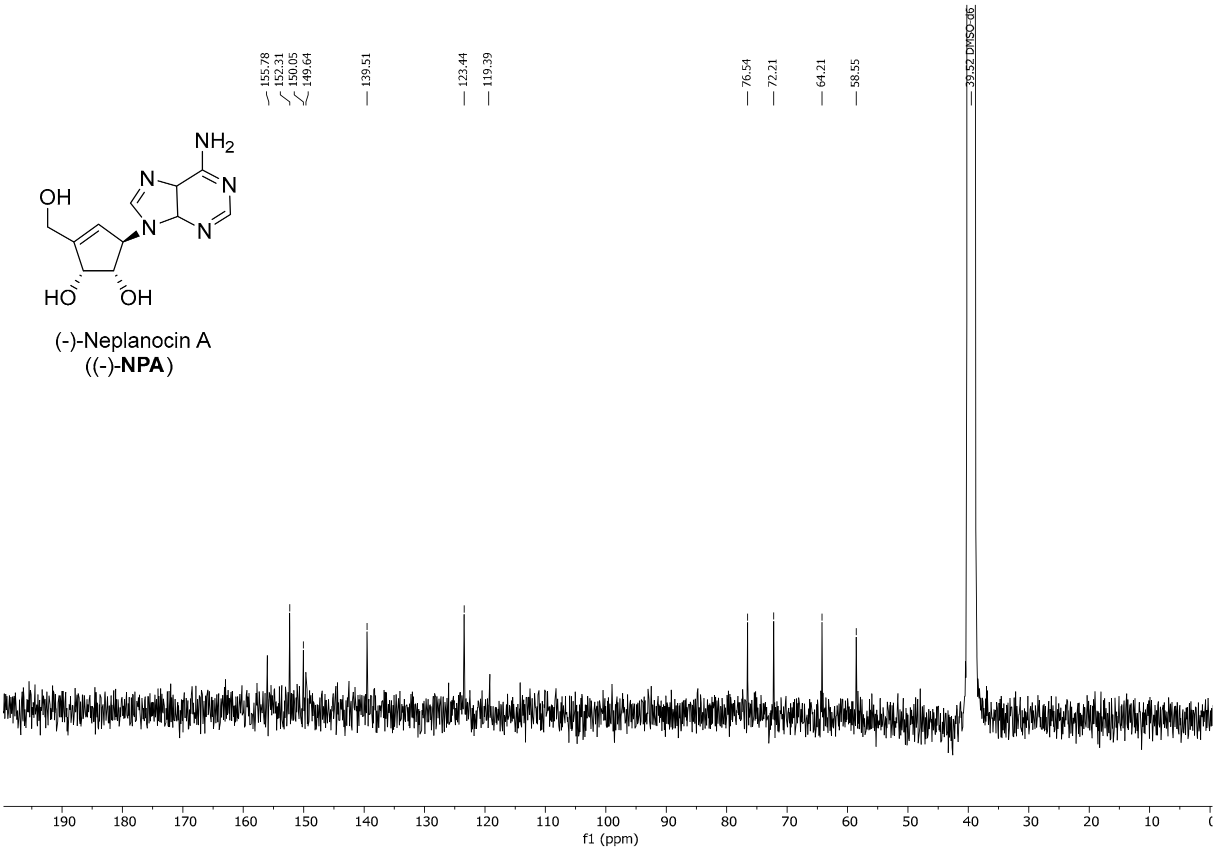

Figure S4. NMR spectra of (-)-NPA.
